# Sarcopenia as a Prognostic Biomarker of Advanced Urothelial Carcinoma

**DOI:** 10.1371/journal.pone.0115895

**Published:** 2015-01-22

**Authors:** Hiroshi Fukushima, Minato Yokoyama, Yasukazu Nakanishi, Ken-ichi Tobisu, Fumitaka Koga

**Affiliations:** Department of Urology, Tokyo Metropolitan Cancer and Infectious Diseases Center, Komagome Hospital, Tokyo, Japan; Fondazione IRCCS Istituto Nazionale dei Tumori, ITALY

## Abstract

**Objectives:**

Sarcopenia, a novel concept reflecting the degenerative loss of skeletal muscle mass, is an objective indicator of cancer cachexia. We investigated its role as a prognostic biomarker in advanced urothelial carcinoma (UC) patients.

**Methods:**

This retrospective study consisted of 88 UC patients with cT4 and/or metastases to lymph nodes/distant organs. Skeletal muscle index (SMI), an indicator of whole-body muscle mass, was measured from computed tomography (CT) images at the diagnosis. Sarcopenia was defined as SMIs of <43 cm^2^/m^2^ for males with body mass index (BMI) <25 cm^2^/m^2^, <53 cm^2^/m^2^ for males with BMI ≥25 cm^2^/m^2^, and <41 cm^2^/m^2^ for females. Predictors of overall survival (OS) were examined using Cox proportional hazard models.

**Results:**

Sixty-seven patients (76%) died during the median follow-up of 13 months. The median OS rate was 13 months. Multivariate analysis revealed that SMI was a significant and independent predictor of shorter OS (hazard ratio (HR) 0.90, *P* <0.001). In the present cohort, 53 (60%) were diagnosed with sarcopenia. The median OS rates were 11 and 31 months for sarcopenic and non-sarcopenic patients, respectively (*P* <0.001). On multivariate analysis, sarcopenia was a significant and independent predictor of shorter OS (HR 3.36, *P* <0.001), along with higher C-reactive protein (CRP) (*P* = 0.001), upper urinary tract cancer (*P* = 0.007), higher lactate dehydrogenase (LDH) (*P* = 0.047), and higher alkaline phosphatase (ALP) (*P* = 0.048).

**Conclusion:**

Sarcopenia, which is readily evaluated on routine CT scans, is a useful prognostic biomarker of advanced UC. Non-sarcopenic patients can expect long-term survival. Evaluating sarcopenia can be helpful for decision-making processes in the management of advanced UC patients.

## Introduction

Increasing evidence supports tumor progression and prognosis depending on not only the biological aggressiveness of the tumor, but also host responses to the tumor, which leads to the wasting and frailty associated with cancer cachexia [1, 2]. Cancer cachexia is a multifactorial syndrome that is characterized by multiple profiles, including weight loss, reduced food intake, and systemic inflammation [3]. Several cancer cachexia-related factors have been investigated to predict survival. C-reactive protein (CRP), a non-specific marker of systemic inflammation, has been identified as a predictor of poor prognosis in patients with various cancers [4]. However, CRP is strongly affected by other diseases, such as infection, cardiovascular disease, and autoimmune disease. This hampers its usefulness in clinical practice because cachexic patients are vulnerable to infection. Weight loss and body mass index (BMI), which are key factors in cancer cachexia, ignore body composition, including fat and lean tissues as well as fluid accumulation such as ascites and edema [5]. Furthermore, the definition of clinically significant weight loss is unclear in the recent setting of increasing obesity [6].

Sarcopenia, a novel concept reflecting the degenerative loss of skeletal muscle mass, has recently been an indispensable element in the definition of cancer cachexia [5, 7]. Sarcopenia is a critical physiological change underlying wasting and frailty caused as a consequence of tumor progression [8]. Thus, sarcopenia, which can be evaluated from computed tomography (CT) images [9], is expected to predict the prognosis of cancer patients. Several studies have revealed the prognostic value of sarcopenia in patients with various cancers, including solid tumors of the respiratory and gastrointestinal tracts, hepatocellular carcinoma, and melanoma [5, 10, 11].

Metastatic and/or locoregionally advanced urothelial carcinoma (UC) patients generally face an unfavorable prognosis. Previous studies demonstrated that the median overall survival (OS) rate was approximately 12–15 months [12, 13]. However, some patients could achieve long-term survival with multidisciplinary treatments [14]. Thus, pretreatment risk assessment based on prognosticators is required to counsel patients about treatment options and participation in clinical trials. In the present study, we investigated the usefulness of sarcopenia as a prognostic biomarker to predict OS in advanced UC patients, along with known prognostic factors such as performance status (PS), anemia, CRP, and the presence of visceral metastasis [13–15].

## Materials and Methods

### Ethical statement

The ethical committee of Tokyo Metropolitan Cancer and Infectious diseases Center Komagome Hospital reviewed and approved the current study protocol (approval number 1420). Written informed consent was obtained from all patients.

### Patients

This retrospective study consisted of 88 UC patients with cT4 and/or metastatic diseases to the lymph nodes and/or distant organs, who were treated at our institution between December 2002 and September 2012. The ethnic group of all patients was Japanese. Pathological diagnoses were made cytologically (25 patients; 28%) and/or histologically using biopsy and/or surgical specimens (63 patients; 72%). The extent of the primary tumor and metastasis was determined from imaging studies, including CT, magnetic resonance imaging (MRI), and/or bone scintigraphy. The following variables were reviewed: age, gender, Eastern Cooperative Oncology Group (ECOG) and Karnofsky PS, BMI, presence of hydronephrosis, primary site (bladder or upper urinary tract), presence of lymph node or visceral metastasis, prior curative surgery, therapies for UC, hemoglobin, white blood cell count, creatinine, albumin, alkaline phosphatase (ALP), lactate dehydrogenase (LDH), corrected calcium, and CRP. BMI was calculated as follows: BMI (kg/m^2^) = ((weight)/(height)^2^). Therapies for UC included any active treatments such as systemic chemotherapy, radiotherapy, and metastasectomy, but not best supportive care. Bajorin’s score was calculated from Karnofsky PS <80% and the presence of visceral metastasis [16].

### Image analysis

CT scans were performed for diagnostic or follow-up purposes. Axial CT images taken within 30 days prior to the initiation of treatment were used for analyses. The third lumbar vertebra (L3) was set as a landmark, and two consecutive slices were selected to measure the cross-sectional areas of skeletal muscle, which were identified using Hounsfield unit thresholds of −29 to +150 [5, 17]. Skeletal muscle at the L3 level included psoas, paraspinal muscles (erector spinae and quadratus lumborum), and abdominal wall muscles (transversus abdominus, external and internal obliques, and rectus abdominus). The mean value of two consecutive images was computed for each patient. The total lumber-skeletal muscle cross-sectional area has been linearly related to whole-body muscle [9]. To evaluate sarcopenia, this value is normalized for stature as is conventional for BMI and body composition components: the skeletal muscle index (SMI) (cm^2^/m^2^) = ((skeletal muscle cross-sectional area at L3)/(height)^2^) [5, 17]. Images were analyzed using Image J 1.47 (National Institute of Health, Bethesda, MD, USA, http://rsb.info.nih.gov/ij). Image analysis was performed by one investigator (H.F.) who was blinded to other variables and patient outcomes.

### Definition of sarcopenia

Appropriate cut-off values of SMI to discriminate OS were evaluated to define sarcopenia in the present UC patient cohort. We finally employed the BMI-incorporated definition of sarcopenia proposed by Martin et al (SMIs of <43 cm^2^/m^2^ for males with BMI <25 cm^2^/m^2^, <53 cm^2^/m^2^ for males with BMI ≥25 cm^2^/m^2^, and <41 cm^2^/m^2^ for females) [6].

### Statistical analysis

Differences in the distribution of variables between groups were evaluated using the chi-square test for categorical variables and Wilcoxon rank sum test for continuous variables. Survival curves were estimated using the Kaplan-Meier method and differences between groups were evaluated using the log-rank test. Univariate and multivariate Cox proportional hazard models tested the associations between variables and OS. Variables with a *P* <0.05 in univariate analysis were entered into multivariate analysis. A reduced multivariate model was generated by backward elimination of the variable with the highest *P* value from each iteration of the multivariate analysis. OS was measured from the date of diagnosis to death or last follow-up. Martingale residual analyses were performed to evaluate the functional form of SMI to be used in a Cox proportional hazard model. All statistical analyses were performed using JMP 9.0.2 (SAS Institute Inc., Cary, NC, USA) and R version 2.14.1 (the R Project for Statistical Computing, Vienna, Austria, www.r-project.org). Statistical significance was defined as two-tailed *P* <0.05.

## Results

### Patient characteristics

Table 1 shows patient characteristics in the present study. The median age was 68 years (range, 39–91 years). Lymph node and visceral metastases were observed in 69 (78%) and 36 (41%) patients, respectively. Our cohort includes 76 patients (86%) who had advanced disease at first presentation and 12 patients (14%) who relapsed after prior curative surgery. Of these 12 patients, one received neoadjuvant chemotherapy and another received adjuvant chemotherapy. Thus, 86 patients (98%) were chemotherapy-naïve at the moment of assessment. After the diagnosis of advanced disease, 67 (76%), 19 (22%), and 5 (6%) patients underwent systemic chemotherapy, radiotherapy, and metastasectomy, respectively. Meanwhile, 10 patients (11%) were treated with best supportive care only. The median (range) SMIs (cm^2^/m^2^) were 41.5 (24.8–64.0) and 34.7 (19.9–55.9) for males and females, respectively.

**Table 1:** Patient characteristics.

**Variables**			**Patients, n (%)**	**Male, n (%)**	**Female, n (%)**	***P* value**
All patients			88 (100)	60 (68)	28 (32)	
Age (years), median (range)			68 (39–91)	66 (39–85)	72 (47–91)	0.044
ECOG PS	0		54 (61)	40 (67)	14 (50)	0.28
	1		25 (29)	14 (23)	11 (39)	
	≥2		9 (10)	6 (10)	3 (11)	
BMI (kg/cm^2^), median (range)			22.1 (16.7–35.9)	22.0 (16.8–29.8)	22.2 (16.7–35.9)	0.58
Hydronephrosis	No		47 (53)	32 (53)	15 (54)	0.98
	Yes		41 (47)	28 (47)	13 (46)	
Primary site	Bladder		42 (48)	28 (47)	14 (50)	0.77
	UUT		46 (52)	32 (53)	14 (50)	
Lymph node metastasis	No		19 (22)	14 (23)	5 (18)	0.56
	Yes		69 (78)	46 (77)	23 (82)	
Visceral metastasis	No		52 (59)	38 (63)	14 (50)	0.24
	Yes	Total	36 (41)	22 (37)	14 (50)	
		Liver	4 (5)	3 (5)	1 (4)	
		Bone	11 (13)	8 (13)	3 (11)	
		Lung	22 (25)	12 (20)	10 (36)	
Prior curative surgery	No		76 (86)	53 (88)	23 (82)	0.44
	Yes		12 (14)	7 (12)	5 (18)	
Therapies for UC	No		10 (11)	7 (12)	3 (11)	0.90
	Yes	Total	78 (89)	53 (88)	25 (89)	
		Systemic chemotherapy	67 (76)	46 (77)	21 (75)	
		Radiotherapy	19 (22)	15 (25)	4 (14)	
		Metastasectomy	5 (6)	3 (5)	2 (7)	
Hemoglobin (g/dl), median (range)			12.6 (3.1–15.8)	12.8 (3.1–15.8)	12.3 (6.2–15.1)	0.040
WBC (10^3^/μl), median (range)			7.6 (2.1–59.0)	7.6 (3.8–59.0)	7.9 (2.1–53.9)	0.82
Creatinine (mg/dl), median (range)			1.0 (0.5–16.0)	1.1 (0.6–16.0)	0.8 (0.5–5.7)	0.002
Albumin (g/dl), median (range)			4.1 (2.7–5.0)	4.1 (2.7–5.0)	4.1 (3.1–4.7)	0.88
ALP (U/l), median (range)			256 (103–1284)	242 (103–1284)	284 (160–572)	0.037
LDH (U/l), median (range)			187 (111–880)	187 (111–681)	187 (118–880)	0.99
Corrected calcium (mg/dl), median (range)			8.7 (7.6–11.0)	8.6 (8.0–11.0)	8.8 (7.6–10.8)	0.43
CRP (mg/l), median (range)			10.0 (0.4–266.0)	9.5 (0.4–266.0)	10.0 (0.4–114.8)	0.79
Skeletal muscle area (cm^2^), median (range)			103.3 (45.0–180.8)	116.1 (66.3–180.8)	76.9 (45.0–127.6)	<0.001
SMI (cm^2^/m^2^), median (range)			40.3 (19.9–64.0)	41.5 (24.8–64.0)	34.7 (19.9–55.9)	0.006
Bajorin’s score	0		52 (59)	38 (63)	14 (50)	0.46
	1		27 (31)	16 (27)	11 (39)	
	2		9 (10)	6 (10)	3 (11)	

Abbreviations: ECOG PS = Eastern Cooperative Oncology Group performance status; BMI = body mass index; UUT = upper urinary tract; UC = urothelial carcinoma; WBC = white blood cell; ALP = alkaline phosphatase; LDH = lactate dehydrogenase; CRP = C-reactive protein; SMI = skeletal muscle index.

### Predictors of overall survival

During the median follow-up of 13 months (range, 1–99 months), 67 patients (76%) died of cancer. The median OS rate was 13 months. Table 2 shows variables associated with OS in univariate and multivariate Cox proportional hazard models. Univariate analysis revealed that age, ECOG PS, BMI, primary site, lymph node metastasis, visceral metastasis, therapies for UC, albumin, ALP, LDH, CRP, and SMI were associated with OS. Multivariate analysis showed that, in a reduced model, SMI was a significant predictor of shorter OS (hazard ratio (HR) 0.90, *P* <0.001), along with upper urinary tract cancer, lymph node metastasis, visceral metastasis, higher ALP, and higher CRP.

**Table 2:** Univariate and multivariate analyses for overall survival.

**Variables**		**Univariate**		**Multivariate (reduced model)**		
		**HR**	***P* value**	**HR**	**95%CI**	***P* value**
Age		1.04	0.005			
Gender	Female	ref	0.63			
	Male	0.88				
ECOG PS	≤1	ref	0.018			
	≥2	2.77				
BMI		0.92	0.015			
Hydronephrosis	No	ref	0.25			
	Yes	1.33				
Primary site	Bladder	ref	0.049	ref		0.001
	UUT	1.63		2.36	1.40–4.03	
Lymph node metastasis	No	ref	0.041	ref		0.007
	Yes	1.89		2.52	1.27–5.40	
Visceral metastasis	No	ref	0.008	ref		0.008
	Yes	1.95		2.09	1.22–3.57	
Prior curative surgery	No	ref	0.74			
	Yes	0.88				
Therapies for UC	No	ref	0.006			
	Yes	0.29				
Hemoglobin		0.94	0.23			
Log WBC		1.92	0.052			
Creatinine		1.09	0.20			
Albumin		0.37	0.005			
Log ALP		3.41	<0.001	2.44	1.15–4.85	0.021
Log LDH		3.31	<0.001			
Corrected calcium		1.43	0.087			
Log CRP		1.33	<0.001	1.25	1.04–1.50	0.018
SMI		0.92	<0.001	0.90	0.88–0.93	<0.001

Abbreviations: ECOG PS = Eastern Cooperative Oncology Group performance status; BMI = body mass index; UUT = upper urinary tract; UC = urothelial carcinoma; WBC = white blood cell; ALP = alkaline phosphatase; LDH = lactate dehydrogenase; CRP = C-reactive protein; SMI = skeletal muscle index; ref = reference.

### Cut-off value of skeletal muscle index to define sarcopenia

**Figure 1:**
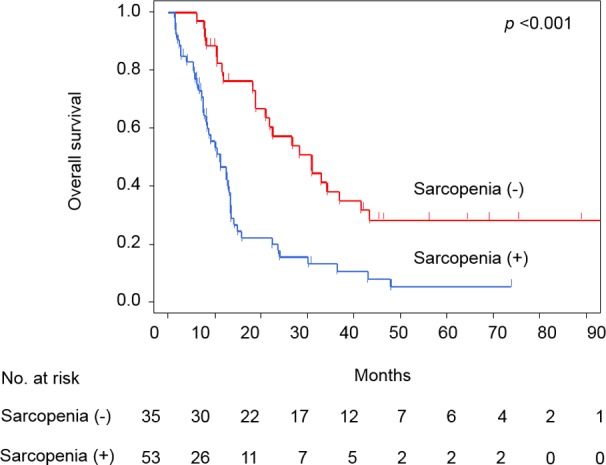
Overall survival of all patients with or without sarcopenia.

We evaluated the optimal cut-offs of SMI below which sarcopenia is defined. On the Martingale residual analyses for cohorts of each sex, the effects of SMI on OS fitted into a linear model and there was no abrupt increase in the HR for OS (S1A Fig. and S1B Fig.), which allows setting the cut-offs of SMI arbitrarily. When the cut-offs were set between 49.1 and 52.4 cm^2^/m^2^ for males and between 35.7 and 40.2 cm^2^/m^2^ for females, the HRs were highest in univariate Cox proportional hazard models (4.54 and 6.63 with *P* <0.001, respectively). Prado’s definition (SMIs of <52.4 cm^2^/m^2^ for males and <38.5 cm^2^/m^2^ for females), which was constructed in a Canadian cohort of 250 patients with respiratory and gastrointestinal tract cancer, was within the ranges of our results [5], and it defines 53 patients (60%) as sarcopenia in our cohort. Recently, Martin et al. reported novel and detailed definition of sarcopenia incorporating BMI (SMIs of <43 cm^2^/m^2^ for males with BMI <25 cm^2^/m^2^, <53 cm^2^/m^2^ for males with BMI ≥25 cm^2^/m^2^, and <41 cm^2^/m^2^ for females) [6]. This definition was constructed in a large cohort of >1,000 cancer patients. According to the Martin’s definition, 53 patients (60%) were diagnosed with sarcopenia in our cohort. The concordance rate between the two definitions was 91% (48/53). In the present study, we employed the Martin’s definition because it is a more sophisticated definition based on a larger cohort and would be generally applicable to various cancer patient populations with different BMI profiles. Fig. 1 shows OS curves according to the presence of sarcopenia in the whole cohort. Overall, the median OS rates were 11 and 31 months for sarcopenic and non-sarcopenic patients, respectively (*P* <0.001).

### Relationships between sarcopenia and other clinical variables

The relationships between sarcopenia and other clinical variables are shown in Table 3. Sarcopenia was associated with lower BMI (*P* <0.001), poorer PS (*P* = 0.001), higher ALP (*P* = 0.002), higher Bajorin’s score (*P* = 0.003), advanced age (*P* = 0.016), visceral metastasis (*P* = 0.017), and lower albumin (*P* = 0.018). There was no association of sarcopenia with hemoglobin or CRP (*P* = 0.095 and *P* = 0.31, respectively).

**Table 3:** Relationships between sarcopenia and other clinical variables.

**Variables**			**Sarcopenia**		***P* value**
			**Yes, n (%)**	**No, n (%)**	
All patients			53 (60)	35 (40)	
Age (years), median (range)			69 (39–91)	64 (47–79)	0.016
Gender	Male		33 (62)	27 (77)	0.14
	Female		20 (38)	8 (23)	
ECOG PS	0		26 (49)	28 (80)	0.001
	1		18 (34)	7 (20)	
	>2		9 (17)	0 (0)	
BMI (kg/cm^2^), median (range)			20.5 (16.7–27.4)	24.0 (17.0–35.9)	<0.001
Hydronephrosis	No		26 (49)	21 (60)	0.31
	Yes		27 (51)	14 (40)	
Primary site	Bladder		28 (53)	14 (40)	0.24
	UUT		25 (47)	21 (60)	
Lymph node metastasis	No		13 (25)	6 (17)	0.40
	Yes		40 (75)	29 (83)	
Visceral metastasis	No		26 (49)	26 (74)	0.017
	Yes	Total	27 (51)	9 (26)	
		Liver	4 (8)	0 (0)	
		Bone	7 (13)	4 (11)	
		Lung	16 (30)	6 (17)	
Prior curative surgery	No		46 (87)	30 (86)	0.89
	Yes		7 (13)	5 (14)	
Therapies for UC	No		9 (17)	2 (6)	0.026
	Yes	Total	44 (83)	34 (97)	
		Systemic chemotherapy	36 (68)	31 (89)	
		Radiotherapy	13 (25)	6 (17)	
		Metastasectomy	1 (2)	4 (11)	
Hemoglobin (g/dl), median (range)			12.3 (3.1–15.8)	12.8 (7.7–15.5)	0.095
WBC (10^3^/μl), median (range)			7.6 (2.1–59.0)	7.5 (3.9–17.6)	0.96
Creatinine (mg/dl), median (range)			1.0 (0.5–16.0)	1.1 (0.6–3.1)	0.48
Albumin (g/dl), median (range)			4.0 (2.7–5.0)	4.2 (3.7–4.7)	0.018
ALP (U/l), median (range)			283 (103–1284)	233 (147–375)	0.002
LDH (U/l), median (range)			197 (118–880)	185 (111–447)	0.11
Corrected calcium (mg/dl), median (range)			8.8 (7.6–10.8)	8.6 (7.9–11.0)	0.059
CRP (mg/l), median (range)			12.0 (0.4–266.0)	6.1 (0.6–85.0)	0.31
Bajorin's score	0		26 (49)	26 (74)	0.003
	1		18 (34)	9 (26)	
	2		9 (17)	0 (0)	

Abbreviations: ECOG PS = Eastern Cooperative Oncology Group performance status; BMI = body mass index; UUT = upper urinary tract; UC = urothelial carcinoma; WBC = white blood cell; ALP = alkaline phosphatase; LDH = lactate dehydrogenase; CRP = C-reactive protein.

### Prognostic significance of sarcopenia on overall survival

Table 4 shows the reduced multivariate Cox proportional hazard model including sarcopenia as a variable. Sarcopenia was the strongest independent predictor of shorter OS (HR 3.36, *P* <0.001), along with higher CRP (HR 1.34, *P* = 0.001), upper urinary tract cancer (HR 2.13, *P* = 0.007), higher LDH (HR 2.12, *P* = 0.047), and higher ALP (HR 2.03, *P* = 0.048).

**Table 4:** Prognostic significance of sarcopenia in multivariate analysis (reduced model).

**Variables**		**HR**	**95%CI**	***P* value**
Primary site	Bladder	ref		0.007
	UUT	2.13	1.24–3.70	
Log ALP		2.03	1.01–3.79	0.048
Log LDH		2.12	1.01–4.11	0.047
Log CRP		1.34	1.12–1.60	0.001
Sarcopenia	No	ref		<0.001
	Yes	3.36	1.90–6.08	

Abbreviations: UUT = upper urinary tract; ALP = alkaline phosphatase; LDH = lactate dehydrogenase; CRP = C-reactive protein; ref = reference.

## Discussion

Sarcopenia is attributed to a decrease in protein synthesis and increase in protein degradation [18]. The decrease in protein synthesis depends on anorexia and the low nutritional status caused by side effects of the treatment and progression of the disease. Meanwhile, the increase in protein degradation is induced by catabolic drivers such as systemic inflammation. Systemic inflammation is induced by cytokines, which are produced by tumor cells or as a result of host responses to tumors, resulting in inflammation-mediated tumor invasion and metastasis [19, 20]. Thus, sarcopenia reflects multiple profiles of cancer cachexia and can be a comprehensive and integrated indicator of cancer cachexia. In the present study, we, for the first time, demonstrated that sarcopenia was a significant and independent predictor of shorter OS in advanced UC patients. Sarcopenia can be readily evaluated through a secondary analysis of diagnostic or follow-up CT images, which is almost universally available in clinical practice [9]. A CT scan is a highly precise modality to estimate human body composition with a reported precision error of 1.4% [21]. Taken together with our results, sarcopenia can be a clinically useful and highly objective prognostic biomarker in advanced UC patients.

Several detrimental effects could occur in cancer patients as a result of sarcopenia. Sarcopenia is associated with chemotherapy toxicities, leading to dose reductions, dose delays, or the termination of chemotherapy [22, 23]. Thus, sarcopenic patients may not fully gain the therapeutic effects of chemotherapy. Furthermore, patients with sarcopenia are also susceptible to infections [24]. Therefore, sarcopenia in itself can contribute to a poor prognosis through these mechanisms in cancer patients.

Sarcopenia reflects many clinical conditions, such as frailty, low nutritional status, active catabolism, and systemic inflammation. Other variables used in the present study also reflected these conditions; advanced age and low PS are related to frailty, while albumin and BMI are indicators of nutrition and catabolism, respectively. Although these parameters were correlated with sarcopenia, they were not significant predictors of OS on multivariate analysis. Given the comprehensive and integrated nature of sarcopenia, it is conceivable that sarcopenia surpasses its confounding factors as a prognostic factor. In the present study, CRP, a marker of systemic inflammation, was not associated with sarcopenia. This may be explained by the non-specificity of CRP as a rapid inflammation marker; it increases in response to various conditions including infection, cardiovascular disease, and autoimmune disease as well as tumor progression [4].

In a contemporary clinical practice setting, the number of overweight or obese patients is increasing [25]. Sarcopenic obesity, the coexistence of obesity and low muscle mass, is considered to be a worst-case scenario due to the combination of two health-related risk factors [26, 27]. The prognostic significance of sarcopenic obesity has recently been confirmed in patients with various cancers [5, 28]. In the present study, sarcopenia was observed in five (28%) out of 18 patients with BMI ≥25 kg/cm^2^, which is the cut-off of obesity for Asian populations [29]. Although three of five patients with sarcopenic obesity died within 12 months, its prognostic value is unclear in the present study probably due to the small number of patients. Further studies in larger patient cohorts are needed to investigate the prognostic role of sarcopenic obesity in advanced UC.

The present study had several limitations. First, sarcopenia can be assessed by other methods, including dual energy x-ray imaging, anthropometry, and bioimpedance analysis [7]. Since a CT scan is necessary for the diagnosis and follow-up of the disease, we used cross-sectional CT imaging. Second, the definition of sarcopenia based on CT imaging has yet to be determined. We sought sex-specific cut-offs of SMI that best predicted OS. Although the volume of skeletal muscle mass differs according to ethnicities [30], the cut-offs defined in a previous Western study were within the cut-off ranges determined in the present study. Third, history of weight loss, a typical symptom of cancer cachexia [7], was not assessed in the present study. Fourth, we must consider the heterogeneity of treatments in our cohort. Although active therapies for UC were adjusted in our multivariate models, the heterogeneity of treatments might bias the present study. In addition, the heterogeneity of treatments in our cohort hampers the assessment of progression-free survival, an important endpoint to select candidates for clinical trials. Histories of treatments including prior curative surgery are also heterogeneous in our cohort; however, prior curative surgery was not associated with the onset of sarcopenia. Finally, the present study was limited by the small number of patients and retrospective study design. Although our results should be confirmed in a larger prospective multi-center cohort, the present study raises the probability that the absence of sarcopenia indicates long-term survival among advanced UC patients.

## Conclusions

Sarcopenia, which is readily evaluated on routine CT scans, is a useful and objective biomarker for predicting the OS of advanced UC patients. Since non-sarcopenic patients can expect long-term survival, the evaluation of sarcopenia can be helpful for decision-making processes in the management of advanced UC patients.

## Supporting Information

S1 Fig Plots of Martingale residuals versus skeletal muscle index and smoothed nonparametric regression curve in males (A) and females (B). (TIF)
