# Supplementary figures and images for: Sarcopenia as a Prognostic Biomarker of Advanced Urothelial Carcinoma
Source: PLoS One. 2015 Jan 22;10(1):e0115895. doi: 10.1371/journal.pone.0115895 (PMC4303429; doi:10.1371/journal.pone.0115895)

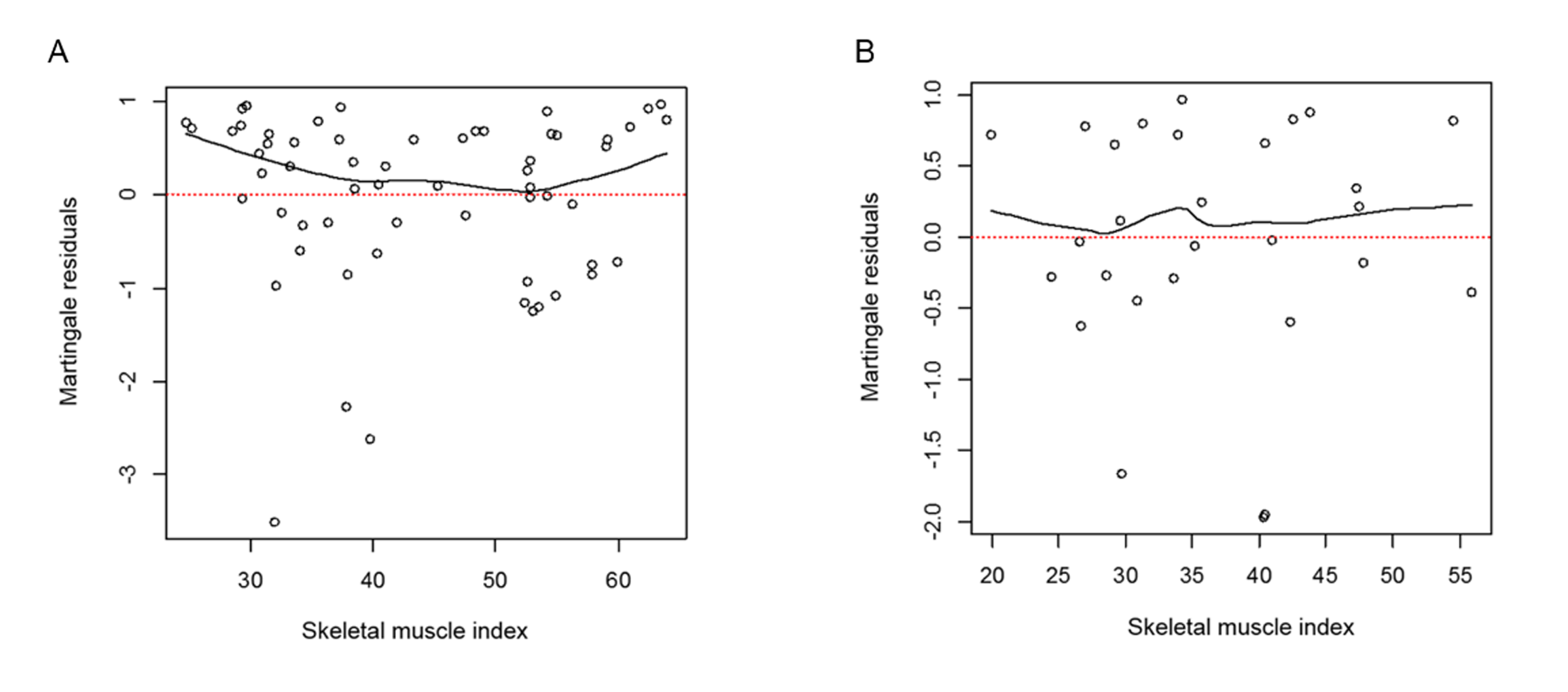

Supplement: S1 Fig — (TIF) [file pone.0115895.s001.tif]
